# Detail computational study about the structural, electronic, optical, and mechanical properties of RbVX_3_ (Cl, Br, I) halide perovskite materials

**DOI:** 10.1039/d3ra03615d

**Published:** 2023-07-28

**Authors:** Naimat Ullah Khan, Javed Iqbal, Abdullah, Ali Algahtani, Jehan Y. Al-Humaidi, Vineet Tirth, Kashif Safeen, Amnah Mohammed Alsuhaibani, Tawfiq Al-Mughanam, Moamen S. Refat, Abid Zaman

**Affiliations:** a Department of Physics, University of Science and Technology Bannu 28100 Pakistan; b Department of Physics, Gomal University DI Khan KP 29220 Pakistan; c Department of Physics, Government Post Graduate College Karak 27200 Pakistan ktkshoraim@gmail.com; d Mechanical Engineering Department, College of Engineering, King Khalid University Abha 61421 Asir Kingdom of Saudi Arabia; e Research Center for Advanced Materials Science (RCAMS), King Khalid University Guraiger, P.O. Box 9004 Abha-61413 Asir Kingdom of Saudi Arabia; f Department of Chemistry, College of Science, Princess Nourah bint Abdulrahman University P.O. BOX 84428 Riyadh 11671 Saudi Arabia; g Department of Physics, Abdul Wali Khan University Mardan 23200 Pakistan; h Department of Physical Sport Science, College of Education, Princess Nourah bint Abdulrahman University P.O. Box 84428 Riyadh 11671 Saudi Arabia; i Department of Mechanical Engineering, College of Engineering, King Faisal University P.O. Box 380 Al-Ahsa 31982 Kingdom of Saudi Arabia; j Department of Chemistry, College of Science, Taif University P.O. Box 11099 Taif 21944 Saudi Arabia; k Department of Physics, Riphah International University Islamabad 44000 Pakistan zaman.abid87@gmail.com

## Abstract

The non-toxic nature of lead-free materials with cubic perovskite structure has attracted the researcher's attention, and huge work is ongoing for the search of such materials. Furthermore, due to demand for their utilization in diverse applications, such as photovoltaic and optoelectronics, these inorganic-halide materials have become more enchanting for engineers. In the present work, all the key properties, including structural, electronic, optical, and mechanical, of rubidium based RbVX_3_ (where X is chlorine, bromine, and iodine) materials were extensively studied *via* first-principle density functional theory (DFT). The study reveals the half-metallic nature of the currently studied materials. For the mechanical stability of RbVX_3_ compounds, all three independent elastic coefficients (*C*_*ij*_) were determined, from which it was concluded that these materials are mechanically stable. Moreover, from the Poison and Pugh's ratios, it was found that the RbVCl_3_ and RbVBr_3_ materials have ductile nature, while RbVI_3_ has brittle nature upon the applied stress.

## Abstract 2

The non-toxic nature of lead-free materials with cubic perovskite structure has attracted the researcher's attention, and huge work is ongoing for the search of such materials.

## Graphical abstract

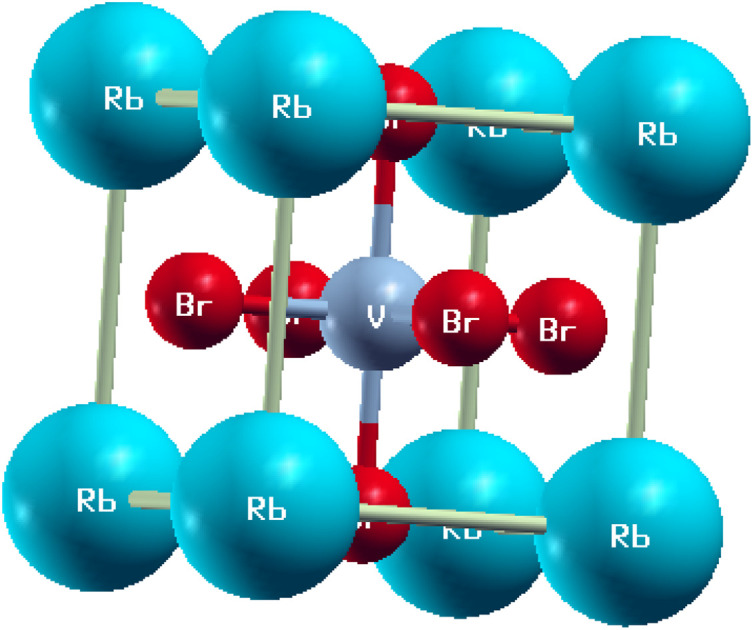

## Introduction

Materials scientists are doing their best to facilitate human society in many aspects of life. Many types of materials have been studied and utilized in different types of applications. Among these varieties of materials, ABX_3_ type (where A and B are cations having larger and smaller ionic radii, respectively, and X is the non-metallic element) materials got special attention due to their employment in a broad range of practical applications such as energy storage, light emitting nano-antennas, wireless-communications, light emitting diodes, fuel cells, superconductors, batteries, photovoltaic electrodes, lasers, and sensing materials.^1–8^ During the last few decades, a lot of such materials have been studied and reported by various researchers either computationally or experimentally for the above-said applications.

These ABX_3_ materials for various types of devices display various combinations of properties depending on the treatment process by the material scientists in the laboratory during their synthesis. There are some ABX_3_ materials exhibiting perovskite structures but their unique nature of behaving like semiconductors in one spin while metallic in the opposite spin makes them ideal for applications such as piezo-electricity, superconductors at high temperature, colossal magneto-resistivity, photo-luminescence, vehicle energy devices, lenses applications, and photovoltaics.^9–17^ In 2002, a research group also investigated computationally such types of materials having dual nature in their spins. The authors reported that both the MTiF_3_ (where M stands for Rb and Cs) materials have metallic nature in their spin-up, while in the spin-down configuration, they showed semiconducting behavior. The authors claimed that both the aforesaid materials are elastically stable and have attractive optical properties.^18^ A lot of other materials have been studied by different research groups using DFT calculations in order to help experimentalists with producing cost effective, easy, and more suitable materials than the existing materials for practical applications.^19,20^

Our research study was also focused on the above-discussed aim, and for that purpose, the structural, electronic, optical, and mechanical properties of the new RbVX_3_ materials have been studied in detail *via* the DFT approach.

## Computational model

In the recent research study, the potentials of TB-mBJ along with the combination of GGA were adopted for various calculations. These potentials are applied in the WIEN2K code and installed with the DFT.^21^ All the important properties, such as structural, electronic, optical and elastic, were studied in the present work. For the aforementioned properties, the *k*-points chosen were 2000. From the optimized energy *vs.* unit cell volume, the basic lattice parameter for the structures was calculated using the well-known Birch–Murnaghan equation of state.^22^ The WIEN2K interface package IRelast was used for calculating the elastic properties of the studied material.^23^ For the optical properties of the presently studied materials, the essential parameters, such as dielectric function, absorption coefficient, optical conductivity, and refractive index, were determined. Some other well-known software like Origin, Xcrysden, and Xmgrace were also used for the plotting purpose in this study. In order to avoid the charge outflow from the atomic spheres, the energy gap between the core and valence electrons was selected as −6 Ry, and also a value of 7 was selected for the RMT × *K*_max_, which is needed for the phenomena of convergence.

## Structural properties

**Fig. 1:**
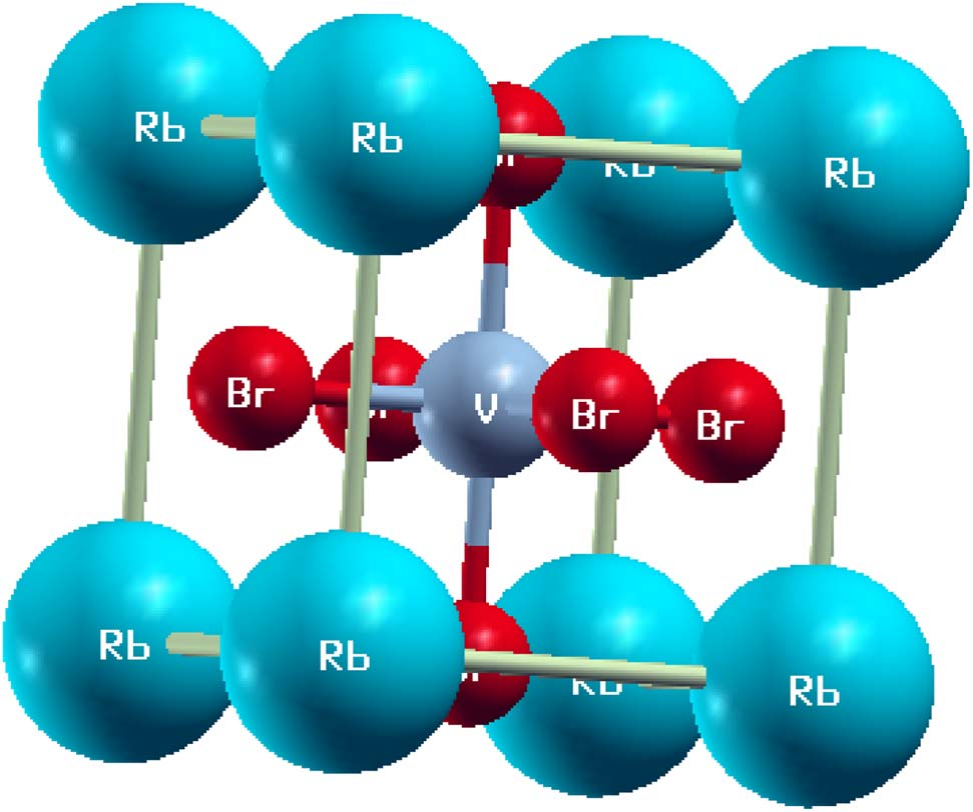
The unit cell structure of RbVBr_3_.

From the structural study, it was concluded that the RbVX_3_ (where X represents Br, Cl, I) material had a cubic crystal structure and is included in the space group of *Fm*3̄*m* (no. 221). Moreover, from this study, it became clear that a total 5 atoms take part in the unit cell of the investigated RbVX_3_ materials, as presented in Fig. 1. The first atom “Rb” take all the corner positions in the cubic unit cell at coordinates (0, 0, 0), and the second atom is vanadium at the B-site, which is at the centered position in the unit cell at coordinates of (0.5, 0.5, 0.5), while the nonmetal X (Br, Cl, I) had the positions of face-centered at coordinates (0, 0.5, 0.5).

**Fig. 2:**
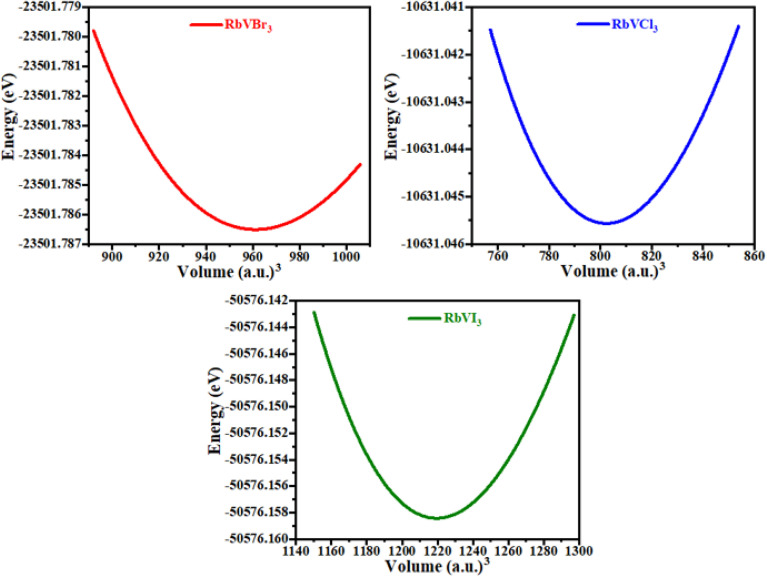
The obtained optimized curves for RbVX_3_ (X = Cl, Br, I) materials.

The following well known formula of the Goldsmith's tolerance factor was followed to find the RbVX_3_ materials structure and thermodynamic endurance (Fig. 2).^24^1
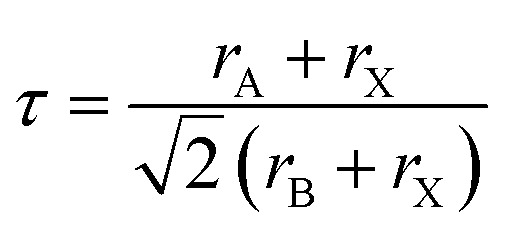


In the above equation, *r*_A_, *r*_B_, and *r*_X_ show the ionic radii of Rb, V, and nonmetal atoms Br/Cl/I, respectively. The calculated *t* values are listed in Table 1 for the RbVX_3_ materials and found in the range of 0.94–0.98, which are needed for the stable perovskite materials, as suggested by Goldsmith.

**Table 1:** The structural parameters of RbVX_3_ (X = Cl, Br, I)

Structural parameters	RbVCl_3_	RbVBr_3_	RbVI_3_
Lattice constant (*a*_o_) (Å)	4.917	5.2224	5.653
Bulk modulus (*B*_0_) (GPa)	42.01	34.11	25.47
Volume at ground state (*V*_0_) (a.u.)^3^	802.351	961.1767	1219.447
Bulk modulus derivative (*B*′)	5.0	5.00	5.000
Ground state energy (Ry)	−10 631.0455	−23 501.786	−50 576.158
Tolerance factor	0.98	0.97	0.94
Formation energy (eV per atom)	−1.8	−1.5	−1.6

From Table 1, it is clear that the optimized cell volumes and lattice constants of the RbVX_3_ materials calculated from the energy-volume optimization curves show variation by replacing the halogen atoms at the X position. Moreover, the unit cell volume and lattice parameters move towards higher values with the replacement of the halogen atom having smaller ionic radii by another halogen atom having relatively larger ionic radii. Among the studied RbVX_3_ materials, the RbVCl_3_ material possesses the smallest lattice constant of 4.917 Å because of the smaller ionic radii of chlorine. However, the lattice constant of the unit cell was recorded as 5.2224 Å for the RbVBr_3_ materials, which is due to the higher ionic radii of Br than Cl. The placement of iodine at position X surges the lattice constant to 5.653 Å, which is due to the higher ionic radii of iodine than chlorine and bromine.^25^ Moreover, the unit cell volumes of the RbVX_3_ materials also showed the same trend of variation as that of the lattice constant with the replacement of the halogen atom at position X. The formation energy of compounds determines the formation and thermodynamic stability of the compounds. The RbVCl_3_ material was found to be more stable due to having the lowest formation energy. The calculated values of formation energy for all the investigated materials are listed in Table 1. The negative values of these compounds show that they are thermodynamically stable and can be synthesized in the laboratory.

## Phonopy

**Fig. 3:**
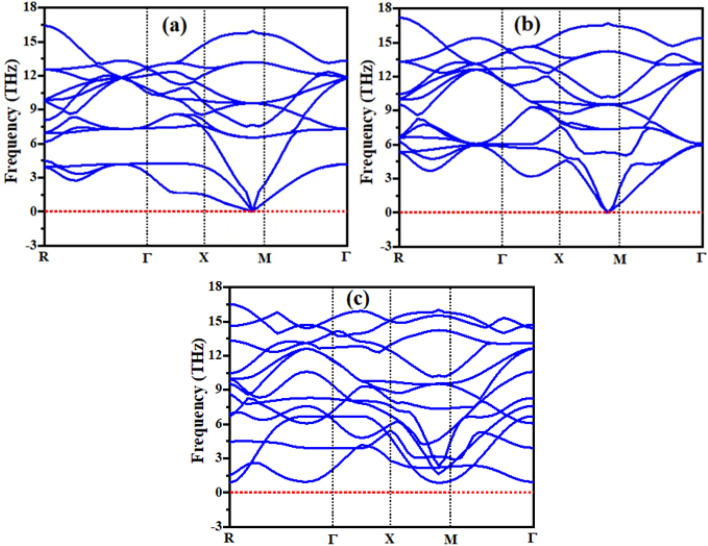
Calculated phonon dispersion curves of (a) RbVBr_3_, (b) RbVCl_3_, and (c) RbVI_3_.

The phonon study of any material is essential because phonons are known as the main factor of the thermal characteristics of any material and their dynamic behavior.^26,27^ In the present study of the RbVCl_3_, RbVBr_3_, and RbVI_3_ compounds, the dispersion band structures of phonon were examined with the help of the WIEN2K package, and their results are presented in Fig. 3. From Fig. 3, it is clear that the curves of the phonon dispersion of all the presently studied materials have positive values, and no values of phonon dispersion curves were detected as negative (*i.e.*, imaginary phonon frequencies), and these values of the phonon dispersion for the investigated materials authenticate the dynamical stability of phonon of all the materials. The same result was also seen for BaAgCl_3_ and BaCuCl_3_ materials in a computational study.^28^

## Electronic properties

**Fig. 4:**
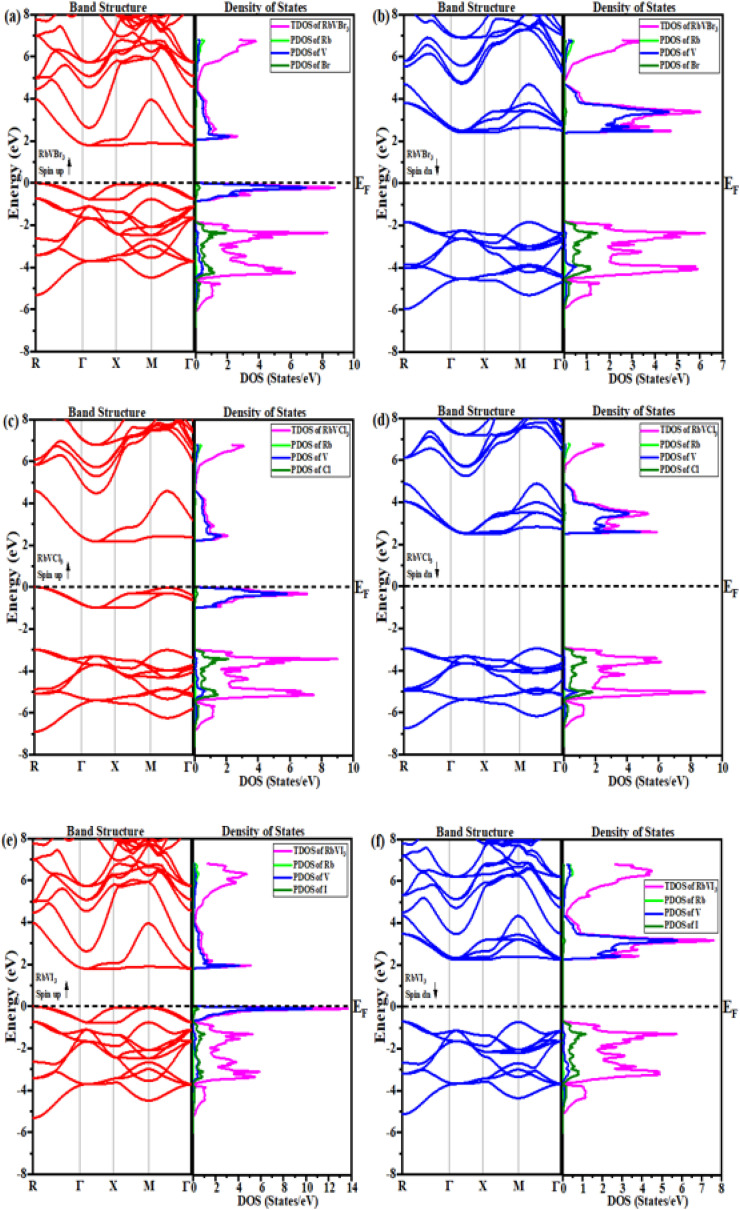
The electronic band structures with the predicted TDOS and PDOS for (a) and (b) RbVBr_3_, (c) and (d) RbVCl_3_ and (e) and (f) RbVI_3_ compounds.

The first step to knowing about any material is to study its electronic properties since all the properties of any material are a function of the electronic state and its behavior. In this study, the electronic properties of the studied RbVX_3_ (where X represents Br, Cl, I) materials were mainly analyzed using the Brillouin zone with high symmetry directions. Using the PBE functional while adopting the Tb-mBJ approximation, the band structures and the respective density of states (DOS) profiles of the RbVX_3_ (where X represents Br, Cl, I) materials were calculated. Fig. 4(a)–(f) illustrates the respective band structures for both the spins of all the three RbVX_3_ (where X represents Br, Cl, I) compounds with the Fermi energy at zero point and the energy band ranges between 8 eV to 6 eV. From the results of the band structures shown in Fig. 4(a)–(f), all the studied compounds showed their metallic nature for the spin-up calculations since the band curves overlap the Fermi level, which is at zero point. But the results for the down-spin calculations for the RbVX_3_ (where X represents Br, Cl, I) compounds were entirely different from the spin-up calculations since none of the band structure curves of the materials cross the Fermi level. The spin-down configuration results of all the presently studied RbVX_3_ (where X represents Br, Cl, I) compounds authenticate their semiconducting nature due to having large band gaps between their valence and conduction bands, as shown in Fig. 4(a)–(f). The TDOS and PDOS patterns of all three studied compounds were also calculated and are presented in Fig. 4(a)–(f). From these results, it is clear that the TDOS and PDOS of “V” in all the RbVX_3_ compounds overlap the zero level for spin-up calculations; however, none of the TDOS and PDOS cross or overlap the zero level for the spin-down configuration. Furthermore, the total and partial density of state outcome strengthens the results of the band structure of the studied materials.

## Mechanical properties

All three independent elastic constants, which comprise all the data regarding the dynamical, thermodynamic, and mechanical activity that also assure the structural stability under strain of all the three RbVX_3_ (X = Br, Cl, I) materials were calculated. These three (*C*_11_, *C*_12_, & *C*_44_) elastic constants were evaluated for the RbVX_3_ (X = Br, Cl, I) compounds and their respective calculated values are listed in Table 2. From the values mentioned in Table 2, it is clear that the RbVX_3_ (X = Br, Cl, I) compounds have positive values of elastic constants and authenticate the conditions of stability that are needed for the perovskite structures as suggested by Spinodal shear and Born criteria (*i.e.*, *C*_11_ & *C*_44_ must be greater than 0, the difference between *C*_11_ & *C*_12_ must be greater than 0, and *C*_11_ + 2*C*_12_ should be greater than 0 for materials having perovskite structures).^29^ Moreover, the Cauchy pressure values were also calculated for the RbVX_3_ (X = Br, Cl, I) compounds while using the elastic constant *C*_12_ and *C*_44_. The values of Cauchy pressure enable us to know about the ductility and brittleness of the materials.^30^ It is clear from Table 2 that the values of the Cauchy pressure calculated for the RbVX_3_ (X = Br, Cl, I) compounds are all positive, hence confirming the ductile nature of all the studied materials. With the utilization of single-crystal elastic constants, the two important moduli, shear modulus *G* and bulk modulus *B*, of the poly-crystalline materials were calculated adopting the Hill approximations. Moreover, for the calculation of Pugh's ratio (*B*/*G*), anisotropy factor, and Young's modulus, the following formulas were employed.^31^2
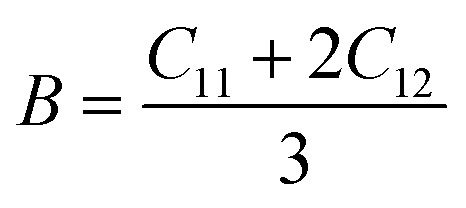
3
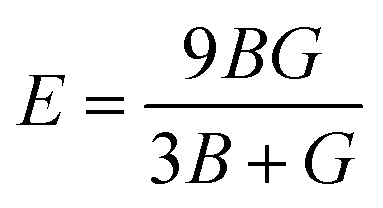
4
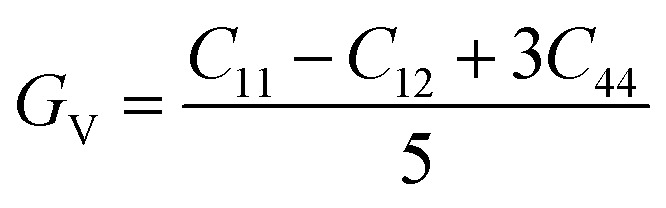
5
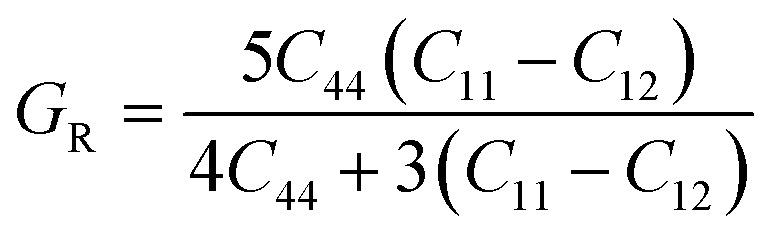
6
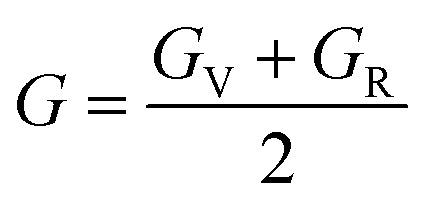
7
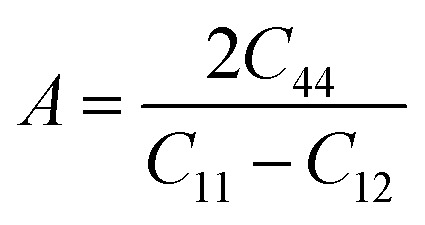


**Table 2:** The elastic parameters of RbVX_3_ (X = Cl, Br, I) materials

Compounds	*C*_11_	*C*_12_	*C*_44_	*B*	*A*	*G*	*E*	*V*	*B*/*G*
RbVCl_3_	49.85	37.81	17.43	41.8	2.8	1.4	4.3	0.71	29.8
RbVBr_3_	63.18	19.46	25.27	34.03	1.15	0.6	0.1	0.74	56.72
RbVI_3_	13.37	32.44	22.016	26	−2.3	38.7	77.7	0.004	0.67

The bulk modulus values calculated for all the three RbVX_3_ (X = Br, Cl, I) compounds were in the range of 26 to 42 GPa, which confirms the soften and non-rigid nature of all the studied materials. Moreover, it is clear from the table that the bulk modulus value of the material having a non-metallic atom with smaller ionic radii at the X position is greater than the other materials having a larger ionic radii non-metallic atom at the X position; the value of bulk modulus moves towards smaller value as the chlorine is replaced by another X atom having comparatively larger ionic radii. Finally, on the basis of the bulk modulus, these materials are best to be utilized in bendable devices because of their softness and flexible nature. Furthermore, the anisotropy factor (*A*) for investigated compounds was calculated and is listed in Table 2. For none of the materials, the anisotropy factor value was seen to be one, which is needed for any material to have isotropic nature, and this validates the anisotropic nature of all the three studied RbVX_3_ (X = Br, Cl, I) materials. The shear modulus *G* values were calculated and found to be the lowest at 0.6 for the RbVBr_3_ material while highest for the RbVI_3_ material. Thus, the RbVI_3_ is more resistive to transverse deformation than the RbVCl_3_ & RbVBr_3_.

Among the three studied materials, RbVCl_3_ and RbVBr_3_ have a greater value of Poisson ratio than 0.33 and confirms their ductile nature, while the RbVI_3_ was found to have brittle nature rather than ductile because it has a Poisson ratio value of 0.004.^32^ The ratio of *B*/*G* as suggested by Pugh's was also calculated and is represented in Table 2. The calculated *B*/*G* ratio values of RbVCl_3_ and RbVBr_3_ are found to be greater than 1.75, while the RbVI_3_ had a lower value of 0.67. So, the Pugh's ratio values of the studied materials also confirm the brittle nature of RbVI_3_ material and ductile nature of the remaining two (RbVCl_3_ and RbVBr_3_) materials.^33^

## Optical properties

**Fig. 5:**
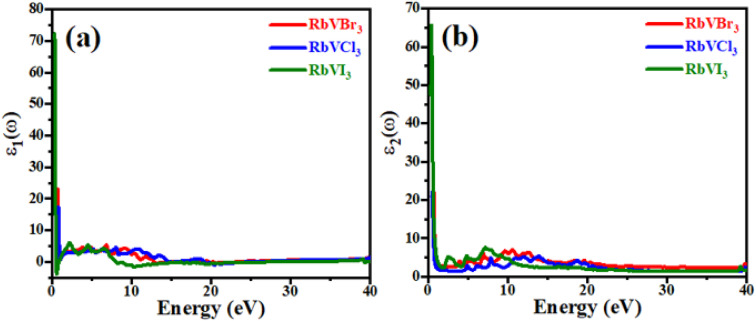
The dielectric function of (a) real part and (b) imaginary part for RbVX_3_ (X = Br, Cl, I).

For any material to be implemented in optoelectronic applications, it is necessary to study the optical response of that material in the numerous electromagnetic energy ranges. The dielectric function that is composed of two main parts (*i.e.*, real *ε*_1_(*ω*) and imaginary *ε*_2_(*ω*) part) of the recently studied RbVX_3_ (X = Br, Cl, I) compounds are presented in Fig. 5. The dielectric function is frequency dependent, and its two parts are related to each other by eqn (8).

8 *ε*(*ω*) = *ε*_1_(*ω*) + j(*ε*_2_(*ω*))

The *ε*_1_(*ω*) parts of the dielectric functions for the recently investigated RbVX_3_ materials in the energy range of 0–40 eV of photons are plotted in Fig. 5(a), and this part explains the polarizability of any material. The static values of the real parts of the RbVX_3_ materials were found as 73 for RbVI_3_, 24 for RbVBr_3_, and 17 for RbVCl_3_, and these curves showed a sudden decline in their values with a very little increment in the photon energy. However, for the further increment in the photon energy than 1.5 eV, the *ε*_1_(*ω*) curves of all the materials showed nearly the same manner of variation, and finally, the RbVI_3_ first achieved the negative value at about 8 eV, which confirms that this material reflects all the incident photons of having energies in this range and behaved like metal.^34^ For the energies of photons greater than 20 eV, all the investigated materials showed no countable variation. The optical band gap and absorption information of any material can be gathered from the imaginary part of the dielectric function. The static value of RbVI_3_ was found to be much higher than the other two studied materials; however, the RbVCl_3_ and RbVBr_3_ had almost similar static values, as shown in Fig. 5(b). All three materials showed a fluctuating manner of increasing and decreasing values in the energy range of 2–22 eV, which indicates the good absorption behavior of these materials in this range. However, for a higher range of photon energy, all three materials showed small peaks of *ε*_2_(*ω*), which means that these materials have less absorption ability in the higher energies of incident photons.

**Fig. 6:**
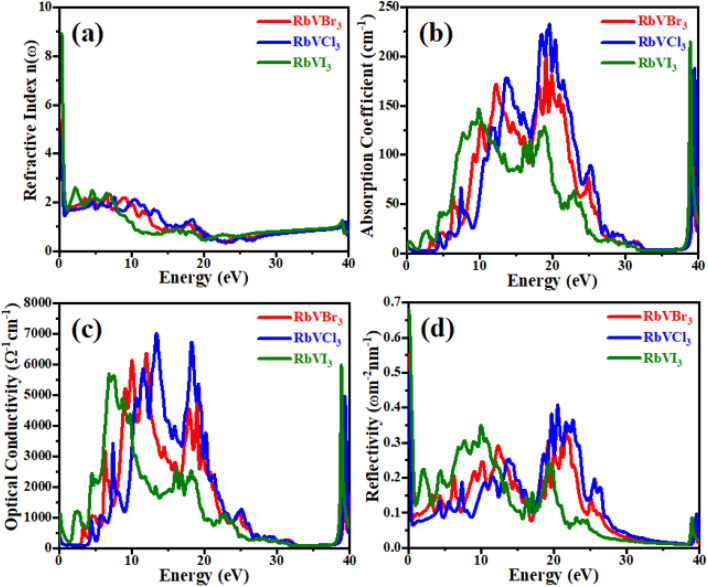
The optical parameters of (a) refractive index (b) absorption coefficient (c) optical conductivity, and (d) reflectivity for RbVX_3_ (X = Br, Cl, I).

The refractive index patterns against the energy values of photons in the range of 0–40 eV of the studied RbVX_3_ (X = Br, Cl, I) materials are presented in Fig. 6(a). The phenomena of dispersion of the photons that are incident on any material can be described by the respective refractive index of that material. The variation in the refractive index curves with varied photon energies was exactly in the same manner as that of the real parts of the dielectric functions of these materials with the photon energies. Moreover, the static values of the refractive index and that of the real parts of the investigated materials fulfilled their well-known relation of *ε*_1_(0) = *n*^2^(0).^35^ From the figure, it is clear that the *n*(*ω*) showed greater variation in its values for the photons having energies in the range of 2–25 eV but almost negligible variation for the photons having energies greater than 25 eV.

Fig. 6(b) and (c) represents the absorption coefficient and the optical conductivity of the presently investigated RbVX_3_ (X = Br, Cl, I) materials in the energy range of 0–40 eV. It is clear from the figure that the curves of both the optical conductivity and absorption coefficient had the same way of improvement and decline in their values with the photon energy variation. For lower values of photon energy, the absorption curves showed small peaks, and as a result, the optical conductivity values were also smaller at these lower ranges of photon energy. However, as the photon energy crosses the limit of 5 eV, the absorption ability of all the materials is enhanced, and as a result, the optical conductivity of the materials is also increased. It was concluded from the patterns of absorption coefficient that the investigated materials had excellent ability of absorption of the incident photons in the energy range of 16–20 eV, and as a result, all the materials showed excess of photo-electrons in these UV energy ranges of incident photons. Moreover, the RbVCl_3_ material showed good ability of the incident photon absorption as well as the emission of photo-electrons in the studied energy range of photons. From the peaks of the absorption coefficient and optical conductivity of the investigated RbVX_3_ (X = Br, Cl, I) materials, it can be concluded that the non-metal having smaller ionic radii at the X position can cause a greater value of absorption coefficient and optical conductivity than the non-metal having greater ionic radii for the same range of incident photons. The highest values of optical conductivity were noted as 7000 unit, 6500 unit, and 5800 unit for the RbVCl_3_, RbVBr_3_, and RbVI_3_, respectively, for the incident UV photon energy range of 5–20 eV. All three materials showed a decline in their values of absorption coefficient and optical conductivity for higher values of incident photon energy, which makes these materials as a reflector for these range of incident photons. The greater values of the optical conductivities and large energy range of absorption of the studied halide materials make them suitable for their use in numerous practical opto-electronic devices.

For the presently investigated RbVX_3_ (X = Br, Cl, I) materials, the reflectivity phenomena against the incident photon energy were also determined in order to know about the surface characteristics of the materials. From Fig. 6(d), it is clear that the peaks of reflectivity show a resemblance with the optical conductivity of the investigated materials; however, the peaks of reflectivity of the investigated materials were not that much sharp like that of the observed peaks of optical conductivity. Since the reflectivity is usually caused by the free electrons at the surface of the materials and increase in the optical conductivity means the generation of excess photo-electrons in the materials, which causes an enhancement in reflectivity from the surface of the materials.

## Conclusion

We reported theoretical results on the structural, electronic, optical as well as elastic properties of RbVCl_3_, RbVBr_3_, and RbVI_3_ by density functional theory using WIEN2K software. These compounds were found stable in a cubic structure. The electronic properties of these compounds indicate that these materials possess dual nature in their spin-up and spin-down configurations. The mechanical properties show that these compounds are mechanically stable. Furthermore, Poisson ratio values deduced from the elastic constants indicate that the compounds RbVCl_3_ and RbVBr_3_ are ductile, while RbVI_3_ is brittle. The greater values of the optical conductivities and large energy range of absorption of the studied halide materials make them suitable for their use in numerous practical opto-electronic devices.

## Conflicts of interest

The authors declare no competing interests.

## Supplementary Material
